# Dual-mode rapid hemostatic materials composed of gelatin and carboxymethyl cellulose for non-compressible bleeding in spinal surgery

**DOI:** 10.1093/rb/rbag135

**Published:** 2026-06-16

**Authors:** Xiaoting Peng, Mingze Ji, Bingcheng Fan, Chuqiang Yin, Ting Wang, Jianyong Du, Guotai Li, Qihui Zhou

**Affiliations:** Qingdao Municipal Hospital, Shandong Key Laboratory of Neurorehabilitation, Shandong Engineering Research Center for Tissue Rehabilitation Materials and Devices, Qingdao Key Laboratory of Smart Rehabilitation Material, School of Rehabilitation Sciences and Engineering, University of Health and Rehabilitation Sciences, Qingdao 266113, China; College of Medicine, Qingdao University, Qingdao 266071, China; College of Medicine, Qingdao University, Qingdao 266071, China; Department of Orthopaedic Surgery, The Affiliated Hospital of Qingdao University, Qingdao 266003, China; Department of Orthopaedic Surgery, The Affiliated Hospital of Qingdao University, Qingdao 266003, China; Qingdao Municipal Hospital, Shandong Key Laboratory of Neurorehabilitation, Shandong Engineering Research Center for Tissue Rehabilitation Materials and Devices, Qingdao Key Laboratory of Smart Rehabilitation Material, School of Rehabilitation Sciences and Engineering, University of Health and Rehabilitation Sciences, Qingdao 266113, China; Qingdao Municipal Hospital, Shandong Key Laboratory of Neurorehabilitation, Shandong Engineering Research Center for Tissue Rehabilitation Materials and Devices, Qingdao Key Laboratory of Smart Rehabilitation Material, School of Rehabilitation Sciences and Engineering, University of Health and Rehabilitation Sciences, Qingdao 266113, China; Qingdao Municipal Hospital, Shandong Key Laboratory of Neurorehabilitation, Shandong Engineering Research Center for Tissue Rehabilitation Materials and Devices, Qingdao Key Laboratory of Smart Rehabilitation Material, School of Rehabilitation Sciences and Engineering, University of Health and Rehabilitation Sciences, Qingdao 266113, China

**Keywords:** spinal surgery, non-compressible bleeding, gelatin, carboxymethyl cellulose, hemostatic material

## Abstract

Spinal surgery, particularly spinal decompression and fusion surgery, commonly encounters intraoperative bleeding. Deep bleeding in the narrow operative area (e.g. bone marrow cavity and venous plexus) is a significant challenge for achieving accurate hemostasis with traditional strategies, posing a life-threatening risk. Herein, a novel dual-form hemostatic material (CMC-Gelatin, CG) composed of carboxymethyl cellulose (CMC) and gelatin is developed for non-compressible hemostasis in spinal surgery. For minor bleeding, CG in powder form reduces blood loss by 69.06% within a mouse bleeding model, demonstrating hemostatic efficacy comparable to that of the commercial Surgicel® powder, while exhibiting superior cytocompatibility. Notably, CG powder rapidly forms an injectable gel upon mixing with saline, making it suitable for severe bleeding in a confined space during spinal surgery. Specifically, in a pig paraspinal microvenous hemorrhage model, CG gel exhibits excellent hemostatic performance, reducing blood loss by 78.44% compared with the untreated group. Importantly, CG demonstrated a comparable hemostasis time and reduced blood loss *in vivo* compared to the commercial Surgiflo®. The designed CG materials exhibit outstanding hemostatic properties in deep, narrow areas, making them a promising hemostatic material in spinal surgery.

## Graphical abstract

**Graphical Abstract:**
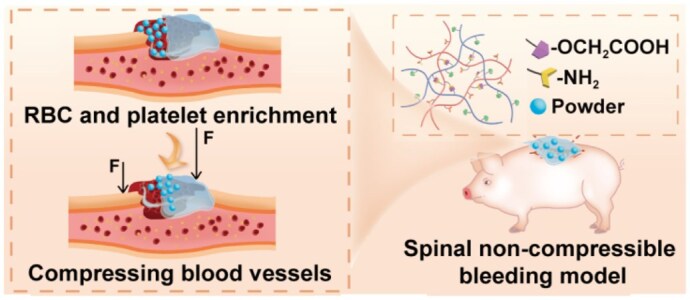

## Introduction

The incidence of spinal surgeries has risen significantly over the past few decades [1]. However, many of these procedures are complicated by uncontrollable intraoperative bleeding due to vascular walls weakened by prolonged compression, intravascular tension, and impaired clotting associated with osteoporosis. These situations may obscure the operative field, elevating the risk of nerve injury and potentially extending the duration of surgery [2, 3]. Furthermore, substantial bleeding in spinal surgery may necessitate perioperative blood transfusions [4], which are associated with various complications, such as anaphylaxis, coagulation disorders, acute lung injury and even sepsis [5]. Conventional hemostatic strategies in clinical practice include tourniquets, electrocoagulation, and direct compression. However, each method has notable drawbacks. For instance, tourniquets and compression may cause complications, such as nerve compression. Electrocoagulation hemostasis often damages surrounding tissues as well as inhibits their healing [6]. Therefore, achieving accurate and effective hemostasis has emerged as a crucial concern in deep, narrow areas of the spine during spinal surgery.

The ideal hemostatic materials, suitable for diverse special occasions and patient types, typically harness multiple mechanisms, such as creating a physical barrier and activating the coagulation cascade [7–9]. However, existing hemostatic materials struggle to address narrow, deep, and irregular non-compressible wounds effectively [10, 11]. They often fail to conform to the wound’s shape, form a robust physical barrier, or apply adequate pressure to the bleeding site, leading to suboptimal hemostatic efficacy. Powder-based hemostatic agents have shown promise owing to their high surface area and exceptional moisture-absorption capabilities, facilitating rapid blood uptake and clot formation [12]. These properties enable rapid blood absorption and facilitate clot formation, thereby effectively controlling bleeding. For instance, Zhang *et al*. [13] developed a hemostatic powder derived from the skin secretions of Andrias davidianus, with efficacy dependent on particle size. Powders are advantageous for their ability to occupy irregular wound spaces and concentrate coagulation factors by absorbing blood. Nonetheless, a significant limitation persists: conventional hemostatic powders lack the mechanical strength needed to control arterial and venous bleeding. Consequently, there is an urgent need for hemostatic powders capable of adhering in a bloody environment [14].

As the complexity of spinal bleeding cases increases and heparin is inevitably used in the treatment plan for occasional but severe spinal lesions [15, 16], there is a need for materials that provide not only effective blood absorption but also enhanced mechanical support [17]. Injectable hydrogels offer the unique advantage of adapting to the intricate contours of deep and narrow wounds, ensuring comprehensive coverage and intimate contact with the bleeding site [18, 19]. Their viscoelastic nature provides the mechanical strength needed to manage even high-pressure bleeding from arterial and venous sources, making them an ideal choice for severe spinal hemorrhage cases [20, 21]. In the context of spinal surgery, advanced hemostatic materials that facilitate a seamless transition from powder to injectable gel form offer a highly versatile solution that can be tailored to meet the unique demands of each case. This combination of powder and injectable hydrogel materials provides a comprehensive strategy for spinal hemostasis across various conditions, effectively controlling bleeding from mild to severe. By leveraging the distinct advantages of both forms, these innovative materials provide robust hemostatic control, ultimately improving patient outcomes.

We aim to develop a dual-form material that could be applied to various stages of bleeding during spinal surgery. The powder form of the material is well-suited for initial blood absorption and control of mild bleeding, while the same composition can also form an injectable gel, providing enhanced mechanical support and comprehensive coverage for severe or difficult-to-access bleeding. Carboxymethyl cellulose (CMC), a carboxylated cellulose derivative, offers cost-effectiveness, excellent water retention, solubility and biocompatibility [22–24]. CMC has been extensively used for hemostasis in combination with other materials [25–28], and CMC-based dressings are known to absorb significant exudates while maintaining a moist environment around the wound to prevent dehydration [29, 30]. Previously, a mechanically robust poly(N-acryloyl 2-glycine) (PACG) supramolecular polymer hydrogel containing amide and carboxyl groups was developed, which enhanced its mechanical properties through multiple hydrogen bonds [31]. The high carboxyl group content on the hydrogel surface enables both tissue adhesion and hemostasis promotion. By incorporating CMC into cryogel preparation, we can harness the benefits of CMC-based wound dressings. Additionally, gelatin, a high-temperature-denatured collagen product, has widespread applications in biomedicine [32, 33]. Gelatin, as a wound dressing, promotes platelet adhesion and aggregation, facilitating blood clotting. At the same time, it supports cell attachment and proliferation [34, 35], making it an ideal biomaterial for hemostasis. We hypothesize that the strategic combination of CMC and gelatin will create a stable hybrid network that synergistically enhances hemostatic efficacy by combining the rapid clotting factor concentration of CMC with the potent platelet recruitment capability of gelatin.

**Figure 1:**
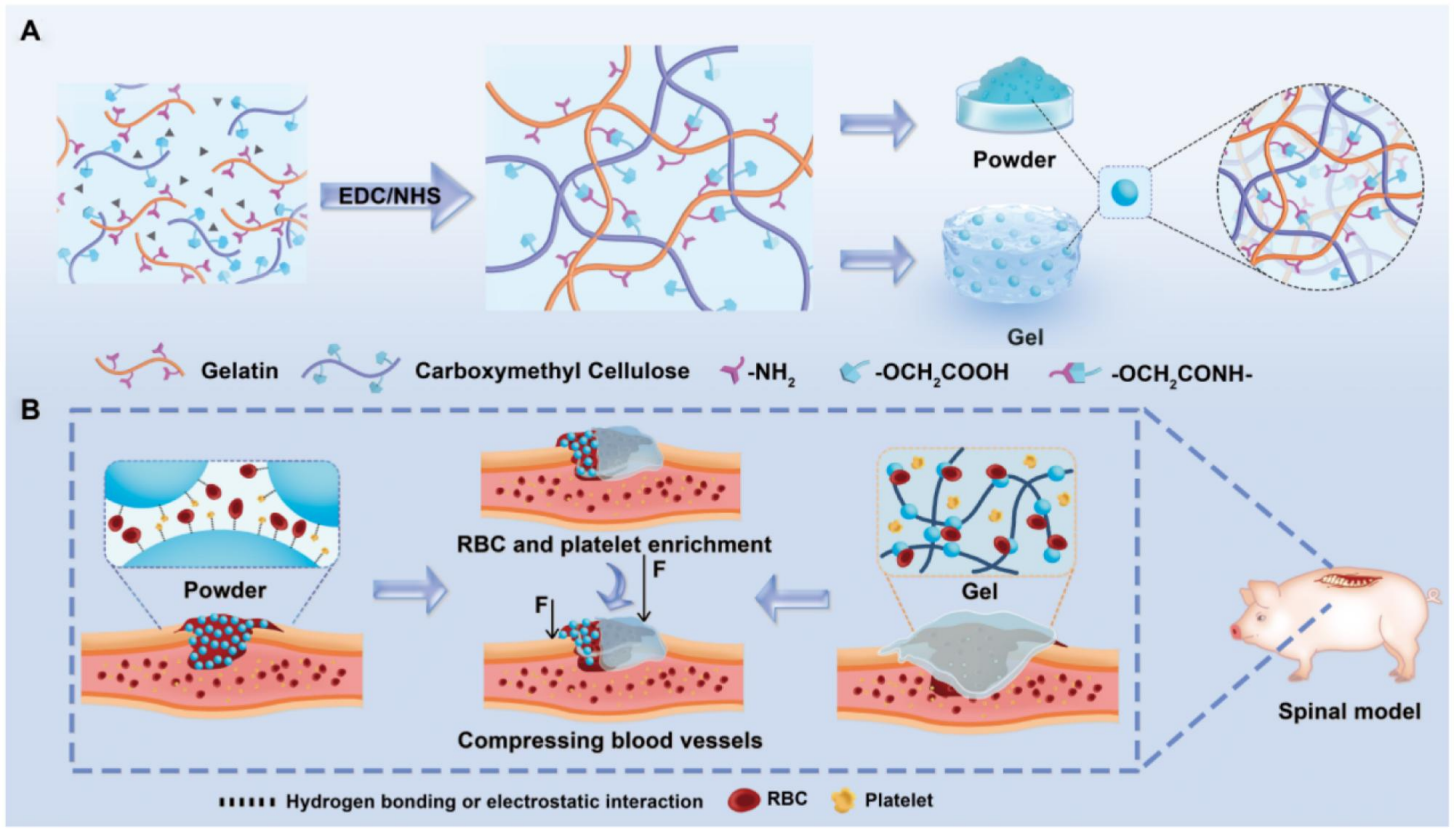
Schematic illustration showing (**A**) the synthesis and preparation of CG powder and its gel, and (**B**) the hemostasis process and mechanism in the porcine spinal hemorrhage model.

In this paper, we report a novel dual-form hemostatic material for non-compressible hemorrhage in spinal surgery. As shown in Figure 1, a composite powder composed of gelatin and carboxymethyl cellulose, synthesized via amidation, was prepared. This powder absorbs blood and strengthens the adhesive barrier on non-compressible wounds. It can also be mixed with saline to form an injectable gel, facilitating rapid hemostasis during minimally invasive spinal surgery. The design of this dual-form hemostatic material combines convenience and cost-effectiveness, making it highly suitable for large-scale production and allowing flexible choice of form based on clinical needs. This novel hemostatic material offers an efficient, safe and economical solution for bleeding control in emergency and surgical settings, especially when rapid, effective management of non-compressible bleeding is required.

## Materials and methods

### Materials and reagents

Gelatin (PharmPure™, gel strength ∼240 g Bloom, G108396-500g), CMC (800–1000 mPa.s, DS = 0.7, average Mw ∼ 200 000 g/mol, DP ∼763, ≥99%, C501052-100g), N-hydroxysuccinimide (NHS, ≥98%, H109330-25g) and 2-morpholino ethanesulfonic acid (2-MES, ≥99%, M163014-100g) were purchased from Shanghai Aladdin Biochemical Technology Co. Ltd. Sodium chloride, sodium hydroxide and disodium hydrogen phosphate dodecahydrate were all analytical grade, purchased from Sinopharm Chemical Reagent Co. Ltd., and were used as received without further purification. 1-(3-Dimethylaminopropyl)-3-ethylcarbodiimide (EDC, ≥98%, N808856-100g) was acquired from Shanghai Macklin Biochemical Co. Ltd. Surgiflo® was from Johnson & Johnson, USA. Surgicel® powder was from Ethicon, Inc.

### Synthesis of CG powder and CG gel

To prepare CG Powder, 1 g of gelatin was dissolved in 50 mL of deionized water at 60°C, with stirring for 10 min to ensure complete dissolution. CMC (0.8 mM) was reacted with EDC (0.8 mM) and NHS (4 mM) for activation in deionized water for 0.5 h, after which the gelatin solution was added and stirred continuously at room temperature for 24 h to form a gel precursor solution. Afterward, Na_2_HPO_4_·12H_2_O (5 mM) and NaCl (0.1 M) were added to the precursor solution, where the pH was adjusted to 8.5 with NaOH solution to form a suspension. Subsequently, the suspension was centrifuged at 5500 rpm for 5 min, and the supernatant was discarded. The obtained precipitate underwent four washes with deionized water to ensure impurities were completely removed. Finally, the purified precipitate was lyophilized and pulverized to obtain the CMC-Gelatin (CG) powder. The formulations were denoted CG30, CG60 and CG90, corresponding to CMC-to-gelatin mass ratios of 30%, 60% and 90% in the dry precursor, respectively. The CG powder was mixed with physiological saline at a mass ratio of 1:4-1:6, as needed, to form a uniform gel. After formulating injectable gels, the same nomenclature was retained for the corresponding formulations to maintain consistency.

### Characterization of CG powder

#### Fourier transform infrared (FTIR) test

FTIR spectroscopy of gelatin, CMC, CG30 powder, CG60 powder and CG90 powder were analyzed using an FTIR spectrometer (Thermo IS5, USA) over the wavenumber range of 4000–500 cm^−1^. Data acquisition was performed by accumulating 32 scans at a resolution of 4 cm^−1^.

#### Scanning electron microscope (SEM) test

The morphology of the powder samples (CG30, CG60 and CG90) was evaluated with a SEM. After grinding and screening through a 60 mesh-150 mesh screen, the powder was mounted on an aluminum stub, gold-sputtered at 10 mA for 90 s using a JEOL JFC-1600 instrument to enhance conductivity, and subsequently imaged at 15 kV.

#### X-ray photoelectron spectroscopy (XPS) test

The elemental composition of the powder samples was analyzed using XPS on a Thermo Scientific K-Alpha+ instrument (USA) with a monochromic Al Kα excitation source at 100 eV. The C 1s, N 1s and O 1s spectra of the samples were processed using Origin 9.1 software with Gaussian-Lorentzian fitting.

### Characterization of CG gel

#### Rheological test

All gel samples (CG30, CG60, CG90) and Surgiflo® were detected by rheometry using a Malvern Kinexus Pro rheometer (UK) with parallel plate geometry, 20 mm diameter and 0.5 mm gap. The measurement procedure commenced with an amplitude sweep to delineate the linear viscoelastic region, followed by a frequency sweep test spanning 0.1–100 Hz. The storage modulus (G′) represents elasticity, whereas the loss modulus (G″) indicates viscosity.

#### Injectability test

The injectability of CG60 gel was evaluated. The injectability assessment was conducted by manually injecting the CG60 gel loaded into a 1 mL syringe barrel, with the entire process visually monitored.

#### Water absorption test

First, 0.3 g (*W*_1_) of each powder sample was mixed with 10 mL of physiological saline for 3 min. Subsequently, the mixture underwent centrifugation at 700 rpm for 5 min, yielding a precipitate that was then weighed (*W*_2_). The water absorption rate was measured using a specific formula:


$$\mathrm{Water}\;\mathrm{Absorption}\;\mathrm{Ratio}\;(\%)=\left(W_{2}-W_{1}\right)/W_{1}\times 100\%$$

### Hemostatic effect *in vitro*

#### Blood clotting time

Blood samples, anticoagulated with sodium citrate, were re-calcified using 0.2 M CaCl_2_ (100 μL per 1 mL of blood) and subsequently introduced into 48-well plates (100 μL per well) containing CG gel (100 μL). Surgiflo® served as the positive control, while wells without gel were used as the negative control. At a predetermined time, 1 mL of normal saline was added to each well, and the diffusion behavior of blood was recorded. The time without blood following the injection of normal saline was defined as the blood clotting time.

#### Blood clotting index (BCI) test

100 µL of re-calcified anticoagulated blood (100 µL 0.2 M CaCl_2_ per 100 µL 3.8% sodium citrate anticoagulated blood) was added to 25 mg powder samples. Surgicel® powder served as the positive control, while plates without powder acted as the negative control. Following incubation at 37°C for 10 min, 1 mL of normal saline was administered to thoroughly lyse the red blood cells that were not incorporated into the blood clot. Subsequently, 100 µL of supernatant from each sample was transferred to 96-well plates for the determination of optical density (OD) at 450 nm. The ratio of BCI was computed by a specified formula:


$$\mathrm{BCI}\;(\%)=A_{\mathrm{sample}}/A_{\mathrm{negativecontrol}}\times 100\%$$

#### Thromboelastography (TEG) test

After injecting 1 mg of powder (CG60 and Surgicel®) into each Eppendorf (EP) tube (1500 μL), 1 mL of phosphate-buffered saline (PBS) was added, then vortexed for 30 s. The resultant mixture was incubated at 37°C for 10 min, after which vortexing was continued for an additional 30 s. The anticoagulated whole blood solution (340 µL), CaCl_2_ solution (20 µL, 0.2 M), and the powder mixture (10 µL) were then added to the test cup, and the whole dynamic coagulation process was monitored by a thromboelastograph meter (CFMS LEPU-8800, China).

### Hemostatic effect *in vivo*

#### Mice liver hemorrhage model

Female mice, aged 5–6 weeks, were randomly assigned to various groups: a negative control group; groups treated with Surgiflo® or Surgicel® as positive controls; and experimental groups treated with CG30, CG60 and CG90 formulations (both in gel and powder forms). Each group consisted of five animals. The mice were anesthetized through intraperitoneal injection of sodium pentobarbital at a dose of 60 mg/kg. Subsequently, the liver was exposed, and a wound measuring 3 mm in length and 2 mm in depth was inflicted on its surface. Immediately, either 300 μL of gel (CG gel and Surgiflo®) or 30 mg of powder (CG powder and Surgicel®) was applied to the bleeding site to ensure a direct comparison under matched practical usage conditions. A pre-weighed filter paper was placed beneath the wound to collect blood, and the amount of bleeding and the time to hemostasis were meticulously recorded. For quantitative analysis, blood loss was determined as the total mass increase of the pre-weighed filter paper used to absorb shed blood. All procedures involving animals adhered strictly to the Guide for the Care and Use of Laboratory Animals and were approved by the Animal Ethics Committee of the University of Health and Rehabilitation Sciences (approval number: 2023-3001).

#### Pig spinal hemorrhage model

Healthy 1-year-old Tibetan pigs weighing approximately 30 kg were selected. CG60 gel and Surgiflo® were used as the experimental and positive control groups, respectively, with a negative control group left untreated. General anesthesia was administered using propofol and sufentanil via tracheal intubation and intramuscular injection. A longitudinal incision centered on the L3/4 extended 2-3 spinous processes upwards and downwards, exposing the root of the transverse processes and the spinal canal to locate the venous plexus on both sides of the spinal canal. For the negative control group, a syringe needle was inserted and the inner wall of the spinal canal was cut to prepare a bleeding model and pre-weighed gauze was quickly placed on the wound. After one minute, the gauze was removed and reweighed to record the amount of bleeding. For the experimental and positive control groups, another side of the vessel or another lumbar vertebra was used for remodeling. After re-puncturing, the wound was immediately covered with the same volume (300 μL) of either Surgiflo® or CG60 gel using a syringe to ensure a direct comparison under matched practical usage conditions. The hemostatic process was repeated and recorded. The mass of the gauze before (*W*_0_) and after (*W*_1_) hemostasis was noted to measure blood loss using the formula:


$$\mathrm{Blood}\;\mathrm{Loss}\;(g)=W_{1}-W_{0}$$

### Biocompatibility *in vitro*

#### Cytocompatibility

The cytocompatibility of the gel was assessed using the Cell Counting Kit-8 (CCK-8) assay and Calcein-AM/PI staining. For the CCK-8 assay, approximately 10^5^ L929 fibroblast cells were cultured in 96-well plates at 37°C for 24 h. Subsequently, the culture medium was replaced with CG60 gel extract solutions, and the cells were incubated overnight. After removing the extraction solutions, 50 μL of CCK-8 detection solution was introduced, and the cells were incubated at 37°C for 1 h under lightproof conditions. The OD was then measured at 450 nm via a spectrophotometer. For live/dead cell staining, approximately 2.4 × 10^5^ L929 fibroblast cells were plated in Transwell-24 plates and cultured at 37°C for 24 h. The prepared CG60 gel was placed in the upper chamber and incubated overnight. After rinsing the chamber 3 times with PBS, a CalceinAM-PI staining solution (2 µM AM and 8 µM PI) was added to each well (200 µL/well), and the cells were cultured at 37°C for 30 min under a light-protected condition. The chamber was rinsed three times with PBS again and photographed under a fluorescence microscope. The cell viability (%) was then calculated from the OD values using the formula mentioned below:


$$\mathrm{Cell}\;\mathrm{viability}\;(\%)=\left({\mathrm{OD}}_{s}-{\mathrm{OD}}_{b}\right)/\left({\mathrm{OD}}_{c}-{\mathrm{OD}}_{b}\right)\times 100\%$$

where OD_*s*_ is the absorbance of the sample containing CG, Surgiflo® or Surgicel® powder, OD_*b*_ is the absorbance of the blank group, and OD_*c*_ is the absorbance of the control group.

#### Hemocompatibility

Fresh whole blood from mice was collected in BD Vacutainer® Evacuated Blood Collection Tubes (BD Vacutainer tubes) containing 3.8% sodium citrate to prevent coagulation. Specifically, 500 μL of anticoagulated blood was diluted with 5 mL of PBS and then centrifuged at 10 000 g for 5 min. Following the centrifugation, the supernatant was discarded, and the resulting pellet was washed repeatedly with PBS until the supernatant became colorless and transparent. The final pellet was resuspended in PBS to a total volume of 10 mL, yielding a 5% (v/v) RBC suspension. To assess the hemolytic activity, powder solutions were prepared in PBS at concentrations of 1 and 3 mg/mL. For each test, 800 μL of the CG powder, Surgiflo®, or Surgicel® powder solution was combined with 200 μL of RBC suspension in separate vials. PBS and 1% (v/v) Triton X-100 solution served as the negative and positive controls, respectively, with 800 µL added to each vial. All vials were cultured at 37°C for 1 h. Following incubation, the samples were centrifuged at 10 000 g for 5 min, and the supernatant was collected for analysis. The absorbance of each supernatant was measured at 540 nm using a UV-visible spectrophotometer (Biotek, PowerWave XS, US). The ratio of hemolysis (%) was then calculated from the OD values using the formula mentioned below:


$$\mathrm{Hemolysis}\;(\%)=\left(A_{s}-A_{p}\right)/\left(A_{t}-A_{p}\right)\times 100\%$$

where *A*_s_ is the absorbance of the sample containing CG powder, Surgiflo® or Surgicel® powder, *A*_p_ is the absorbance of the PBS group, and *A*_t_ is the absorbance of the positive Triton X-100 group.

### Biocompatibility *in vivo*

#### Rats subcutaneous degradation model

CG60 gel was sterilized with UV illumination overnight for subsequent implantation experiments. We randomly selected male Sprague Dawley (SD) rats and removed hair from both sides of the spine. Two implants of the sterilized gel were subcutaneously injected on each side of the spine. At 2, 4 and 6 weeks post-implantation, major organs (heart, liver, spleen, lung and kidney) were harvested for Hematoxylin and Eosin (H&E) staining, and the residual gel mass was quantified simultaneously.

#### Pig spinal hemorrhage model

At postoperative day 48, lumbar vertebrae adjacent to the surgical site and those in normal areas were extracted for H&E staining.

### Statistical analysis

The statistical data were presented as mean values ± standard deviation (SD). The sample size for each experiment is indicated in the corresponding figure legends. One-way ANOVA, coupled with Tukey’s test, was employed for multiple comparisons of variance, as specified in the text. *P *< 0.05 indicates that the difference is statistically significant. The statistical results were visualized using GraphPad Prism 8.0 (GraphPad Software, USA) and OriginPro 2021.

### Medical ethics

All *in vivo* experiments on animals were conducted with the formal approval of the Animal Ethics Committee of the University of Health and Rehabilitation Sciences (KFDX: NO.2023-3001) and in accordance with the guidelines of the Institutional Animal Use Committee of China.

## Results and discussion

### Preparation and characterization of the CG hemostatic powder/gel

**Figure 2:**
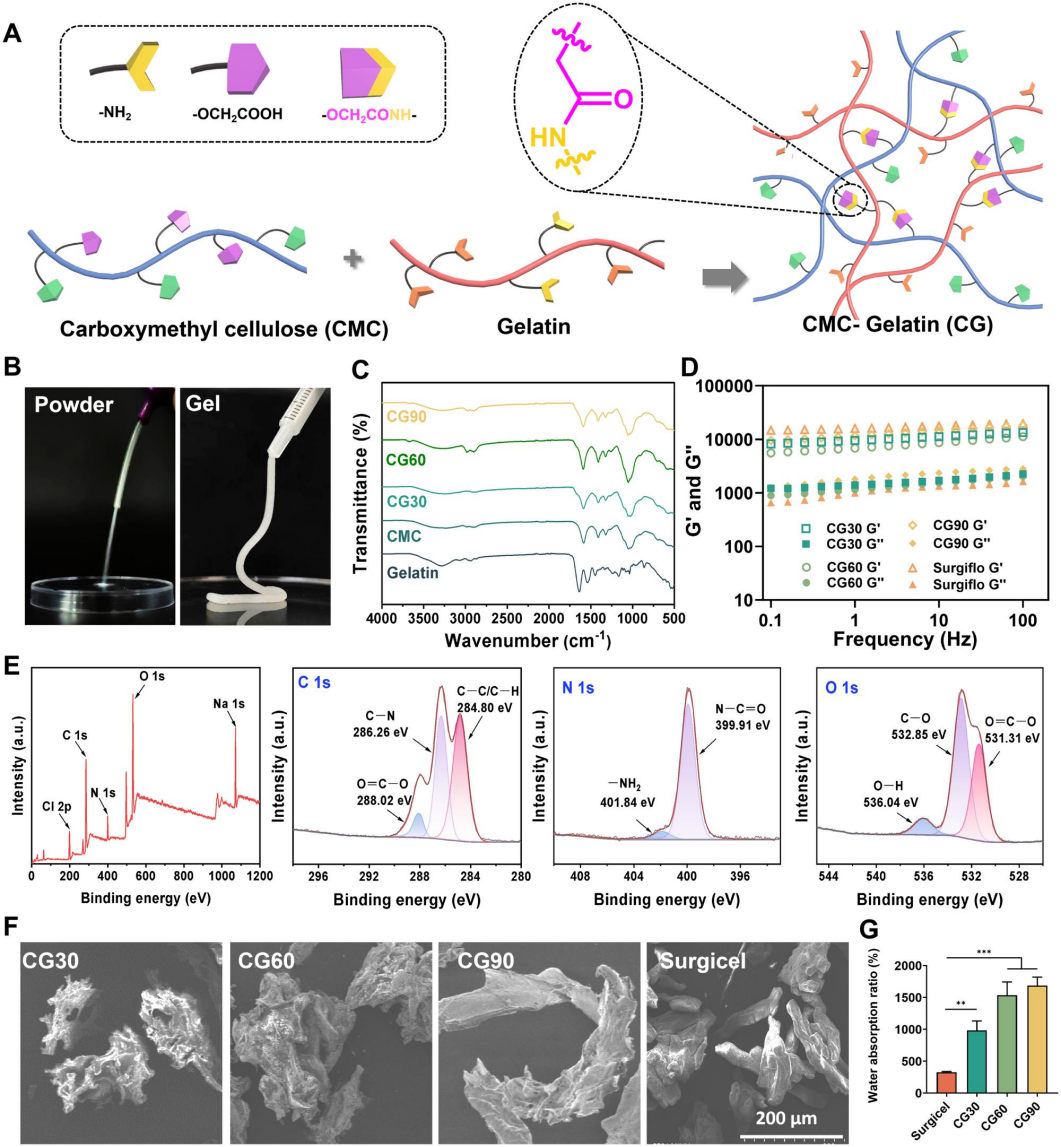
Preparation and characterization of the CG hemostatic powder and gel. (**A**) Synthesis of CG. (**B**) Photos of CG powder and its gel. (**C**) FTIR spectra of CMC, gelatin, and CG synthesized with different ratios of CMC and gelatin. (**D**) Rheological curves of the CG gels and Surgiflo® under frequency sweep mode. (**E**) XPS high-resolution spectra: C 1s, N 1s and O 1s peaks of CG60. (**F**) SEM images of the CG powders with different ratios of CMC/gelatin and Surgicel®. (**G**) The water absorption ratio of all CG powder synthesized using different ratios of CMC/gelatin. Data are presented as mean ± SD. **P < 0.01, ***P < 0.001, ns: not significant.

The CG was synthesized by grafting gelatin onto CMC using EDC and NHS, as illustrated in Figure 2A. Two distinct forms of hemostatic materials were developed to cater to various hemostasis applications in spinal surgery. Hemostatic powder was prepared by freeze-drying the cross-linked CG products. This white powder can be uniformly dispensed through a nozzle (Figure 2B). By adding saline to the hemostatic powder, a homogeneous and continuous semi-transparent hemostatic gel with excellent injectability was obtained. The CG powder could be mixed with physiological saline to form a uniform injectable gel within approximately 30 s. The FTIR spectra of gelatin, CMC, and CG products were acquired and presented in Figure 2C. In the FTIR spectrum of all CG products, characteristic peaks of both gelatin and CMC were evident. Furthermore, the presence of a new peak at around 1700 cm^−1^, assigned to the amide stretching vibration, indicated successful cross-linking between gelatin and CMC. Moreover, the CG products showed retained carboxyl peaks at about 3275 cm^−1^ and 1585 cm^−1^, indicating the presence of free carboxyl acid groups. To further confirm the content of the residual carboxyl groups, we performed zeta potential measurements. As shown in Supplementary Figure S1, all of the CG gels had the negative charges brought from the carboxylic acid group on the surface. Interestingly, CG60 had the most abundant negative charges among the three. This suggests that the surface negative charge was not determined solely by the total CMC content, but also by the balance between residual carboxyl-group retention and their interfacial accessibility. In CG90, the higher polysaccharide fraction may promote stronger intermolecular association and partial shielding of carboxyl groups within the network, whereas CG60 may provide a more favorable composition for exposing ionizable carboxyl groups at the material surface. These results confirmed the presence of abundant free carboxyl groups, which might facilitate tissue adhesion, platelet aggregation and blood coagulation [36]. The negatively charged surfaces of CG materials can activate factor XII upon contact with blood, a hallmark of the intrinsic pathway. This activation triggers a cascade leading to thrombin generation and fibrin clot formation. Additionally, the carboxyl groups can bind to Ca^2+^, an essential component of the intrinsic coagulation pathway. Thus, the CG materials may have the potential to initiate the intrinsic pathway. The cross-linking between gelatin and CMC in CG60 was further confirmed through XPS analysis. As depicted in Figure 2E, the N 1s spectrum exhibited prominent -NH_2_ and amide peaks at 401.84 eV and 399.91 eV, providing evidence for the formation of CG, and confirming the successful cross-linking between gelatin and CMC.

As a hemostatic material to prevent bleeding during spinal surgery, the CG gel should have strong mechanical properties to serve as a physical barrier [37]. The results, as depicted in Figure 2D, revealed that the elastic modulus (G′) of all CG hemostatic gels was approximately six times that of the viscous modulus (G″), indicating the gels’ ability to maintain their structure due to their excellent mechanical properties. Compressive modulus tests were performed to further evaluate the mechanical properties of CG gels. The results demonstrated that the CG60 gel possessed excellent compressive strain and stress compared to the CG30 and CG90 (Supplementary Figure S2). Specifically, CG60 achieved a maximum compressive strain of 105% while sustaining a peak stress of 106 kPa, whereas Surgiflo® showed a maximum strain of 103% with a peak stress of 93 kPa. These data highlight that CG60 showed improved mechanical properties, which are critical for maintaining hemostatic efficacy in dynamic surgical environments, such as spinal procedures involving pulsatile bleeding. The hemostatic performance of CG powder was found to be critically dependent on its microstructure [38]. To investigate this relationship, SEM was employed to analyze the surface morphology of CG powder in comparison to commercially available Surgicel® powder. As illustrated in Figure 2F, CG powder displayed distinct microscale protuberances and surface asperities, contrasting with the relatively smooth topography observed in Surgicel® powder. The increased specific surface area offers additional adhesion sites for platelets, thereby facilitating hemostasis. Moreover, a hemostatic dressing with effective tissue fluid and blood absorption capabilities not only elevates the concentration of coagulation components at the bleeding site to facilitate coagulation but also maintains wound moisture [39, 40]. To verify the absorption performance of the CG powder, the powder was added to normal saline, and the solution was observed to exceed its absorption capacity for 3 min. After low-speed centrifugation, the supernatant was removed, and the remaining gels were weighed. The water absorption ratios of CG 30, CG60 and CG90 gels were 983 ± 150%, 1536 ± 208% and 1688 ± 133%, respectively (Figure 2G), confirming that the designed gels exhibit excellent liquid absorption capacity and are suitable for treating hemorrhage. Additionally, CG materials exhibited much higher water absorption capacity than Surgicel® (329 ± 10%), enabling rapid absorption of blood and tissue fluids at the wound site. This was also consistent with its microscale protuberances and surface asperities as observed in Figure 2F.

### Biocompatibility and hemostatic effect *in vitro*

**Figure 3:**
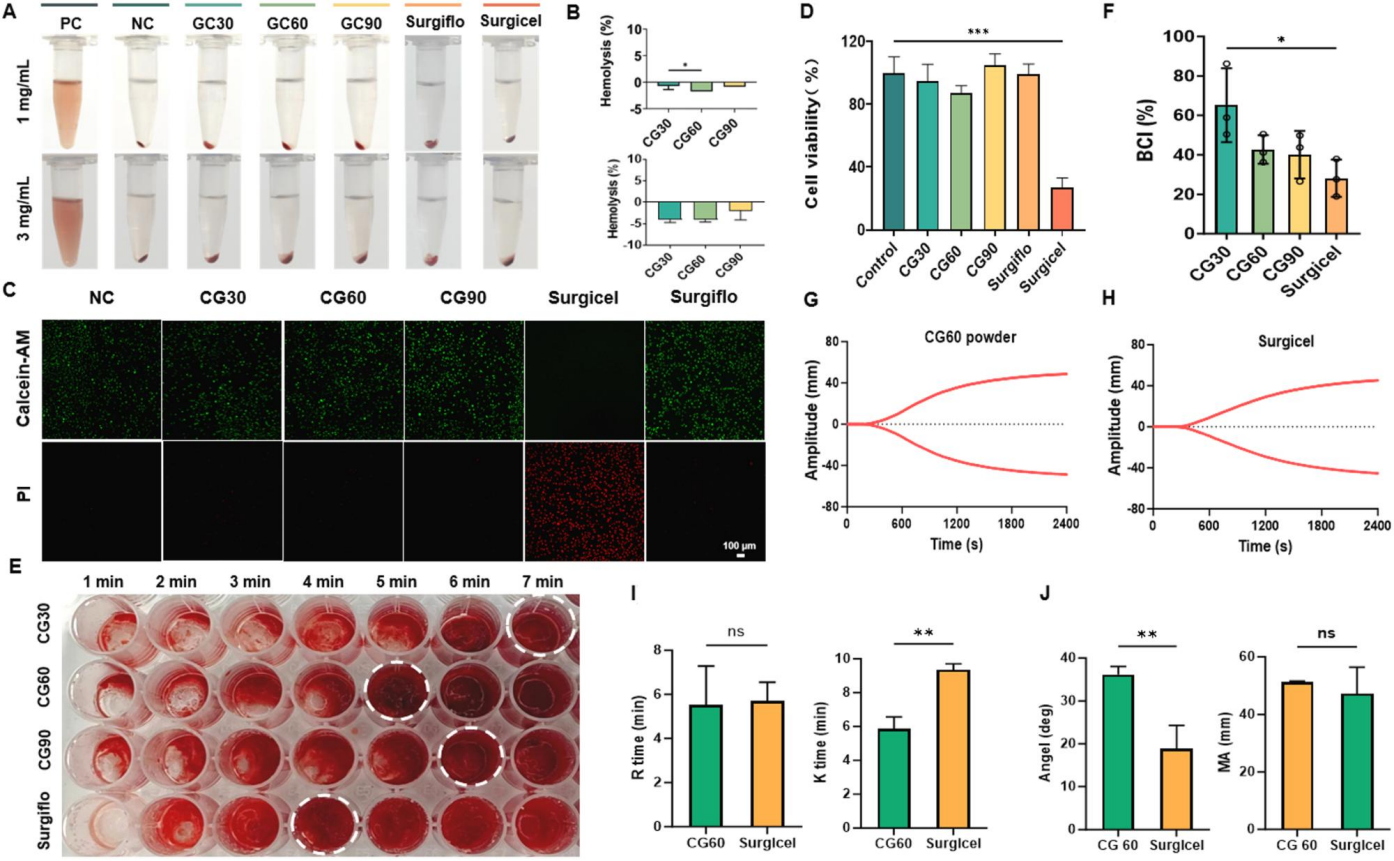
Biocompatibility and hemostatic effect *in vitro*. (**A**) Optical images of hemolysis test *in vitro* (PC: 0.1% Triton X-100, NC: PBS) (*n* = 3). (**B**) Hemolysis ratio of CG30, CG60 and CG90. (**C**) Live/dead staining for L929 cells contacting with CG30, CG60 and CG90 for 1 day. Scale bar: 100 µm. (**D**) The effect of CG30, CG60 and CG90 on the proliferation of the L929 cells (*n* = 3). (**E**) Blood clotting time of CG30, CG60, CG90, and Surgiflo®; the white dashed circle indicates complete coagulation. (**F**) BCI of CG powder and Surgicel®. (**G**) TEG of CG60 powder and (**H**) Surgicel®. (**I**) R and K time of CG60 powder and Surgicel®. (**J**) Angle and MA of CG 60 powder and Surgicel®. Data are presented as mean ± SD. *P < 0.05, **P < 0.01, ***P < 0.001, ns: not significant.

Evaluation of *in vitro* biocompatibility, as a key biological characteristic of materials, was imperative in biomaterial research [41, 42]. The hemolytic test (Figure 3B) revealed a hemolysis ratio of less than 5% in all groups (*P *> 0.5), indicating good blood compatibility. Optical images (Figure 3A) further corroborated this finding, demonstrating that all experimental groups exhibited a color comparable to that of PBS. This observation indicated no erythrocyte membrane rupture, which was consistent with the quantitative data obtained. These results indicated satisfactory *in vitro* hemocompatibility of the powders. For cytocompatibility assessment, the CCK-8 assay and live/dead staining were employed. The CCK-8 assay results (Figure 3D) demonstrated that, except for the Surgicel® powder group, the cell proliferation capacities in the extract-treated groups were comparable to those of the untreated control group, with all cell viability rates exceeding 85%, indicating favorable cytocompatibility. As shown in Figure 3C, L929 fibroblasts cultured for 24 h in extracts of CG30, CG60, CG90 and Surgiflo® exhibited a vibrant status with a typical long-spindle shape morphology. The proportions of dead cells in the CG30, CG60, CG90 and Surgiflo groups were similar to those in the control group. In contrast, the Surgicel® powder group displayed extensive areas of dead cells, consistent with the CCK-8 results. All results demonstrated that the CG materials possess good cytocompatibility, which is substantially superior to that of the commercial product Surgicel® powder.

To further assess the CG’s *in vitro* coagulation capabilities, the clotting time of whole blood in a 48-well plate was measured (Figure 3E). Compared with the control group (∼10 min), all CG groups significantly accelerated blood coagulation. With the addition of CG30, CG60 and CG90, the whole-blood clotting time was significantly reduced to approximately 7, 5 and 6 min, respectively. We also calculated the blood coagulation index (BCI) to quantitatively evaluate the hemostatic performance of all CG powder and Surgicel® powder. As shown in Figure 3F, after 10 min of treatment, the BCI of the CG60 and CG90 powder groups were 42.68% and 40.07%, respectively, demonstrating comparable efficacy to the Surgicel® group without statistical significance (*P *> 0.05). These results suggested that both CG60 and CG90 significantly accelerated blood coagulation. In the follow-up experiment, the CG60 group with the better coagulation effect was selected for the following experiment. To further investigate blood coagulation dynamics in real time, the TEG tests were performed (Figure 3G–J). The results demonstrated that compared to Surgicel®, the reaction (R) time of CG60 powder was similar, but the kinetics (K) time was shorter (Figure 3I), indicating that its coagulation initiation time was close, but fibrin formation was more rapid and suggesting a potentially enhanced coagulation response. Moreover, CG60 powder achieved a larger angle and higher maximum amplitude (MA) (Figure 3J), highlighting its superior ability to accelerate clot growth and enhance mechanical stability. Importantly, CG60 powder also showed statistically significant improvements in K time and angle over Surgicel®, further validating its enhanced hemostatic performance. Taken together, all developed hemostatic materials demonstrated sufficient biocompatibility and hemostatic efficacy for subsequent *in vivo* experiments. Considering its overall balance of cytocompatibility, hemocompatibility, rapid clotting performance, relatively low BCI, favorable TEG parameters, and suitable gel properties for injectable use, CG60 was selected as the optimized formulation for subsequent *in vivo* studies.

### Hemostatic effect in the mouse bleeding model

**Figure 4:**
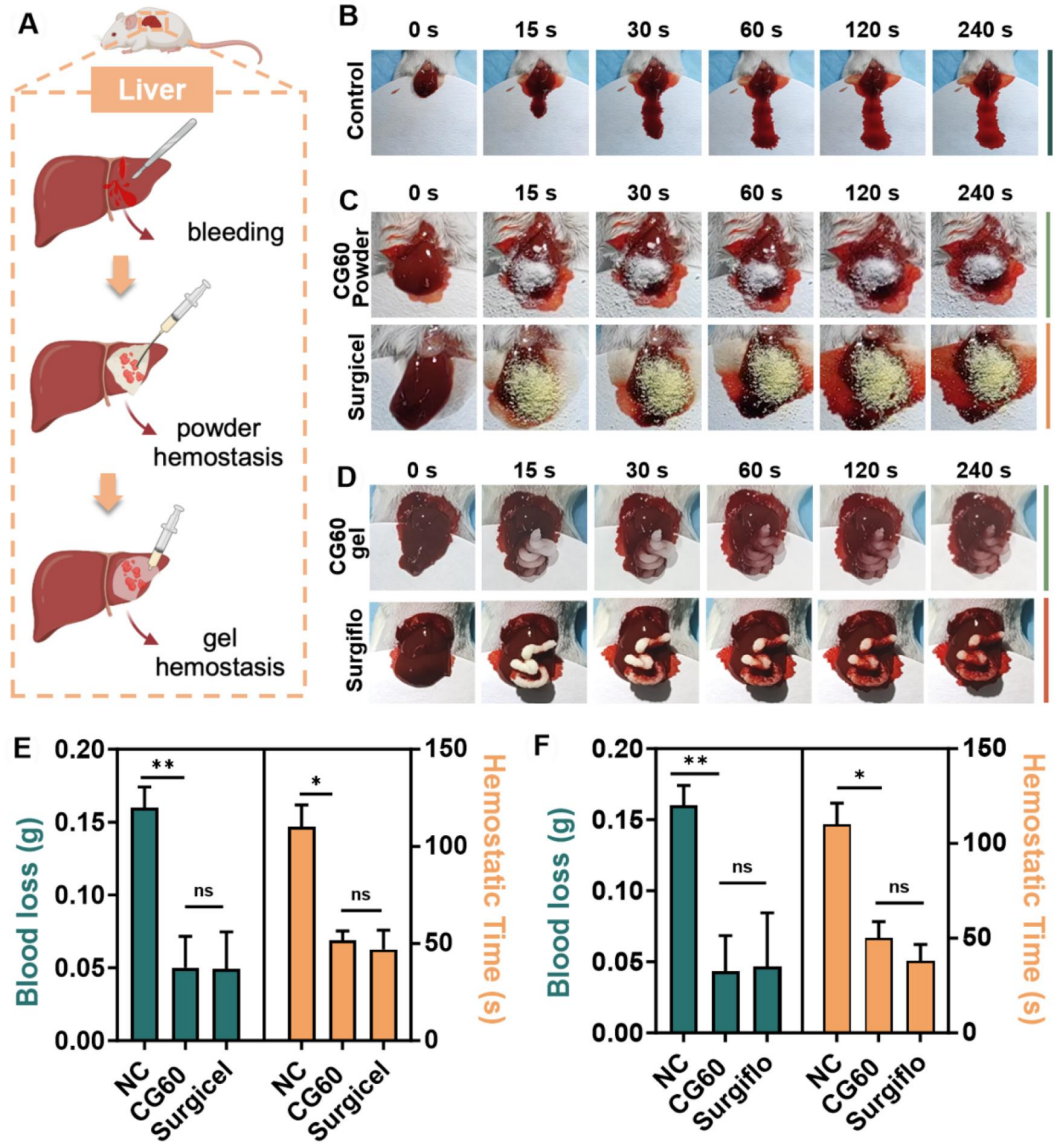
Hemostatic effect in the mouse bleeding model. (**A**) Schematic diagram of the mouse liver model and the hemostat application of CG60 gel and powder. (**B**) Photos of the hemostasis process in the blank, (**C**) CG60 powder, Surgicel® and (**D**) CG60 gel and Surgiflo® groups (*n* = 5). (**E**) Blood loss and hemostatic time of CG60 powder. (**F**) Blood loss and hemostatic time of CG60 gel. Data are presented as mean ± SD. *P < 0.05, **P < 0.01, ns: not significant.

To evaluate the hemostatic efficacy of CG60 gel and powder, the mouse bleeding model was initially utilized. In the hemostatic powder group, commercially available oxidized regenerated cellulose hemostatic powder (Surgicel®) served as the positive control (PC), whereas in the hemostatic gel group, absorbable hemostatic fluid gel (Surgiflo®) was selected as the PC. The macroscopic hemostatic effect was depicted in Figure 4C and D, where CG60 gel and its powder demonstrated hemostatic effects comparable to those of Surgiflo® and Surgicel®, as evidenced by the bloodstains observed on the filter paper. This finding was further quantitatively confirmed by assessing the blood loss (Figure 4E and F). Notably, the CG60 group showed no significant difference compared to the PC group. Regarding hemostasis time, the CG60 powder, CG60 gel, Surgicel® and Surgiflo® groups exhibited significantly reduced hemostasis times of 51.5 ± 4.9 s, 50 ± 8.7 s, 47 ± 9.9 s and 38 ± 8.7 s, respectively, compared to the NC group (110 ± 11.3 s) (Figure 4E and F). Similarly, no significant differences were observed between the CG60 and PC groups.

### Hemostatic effect on non-compressible hemorrhage in pig spinal surgery

**Figure 5:**
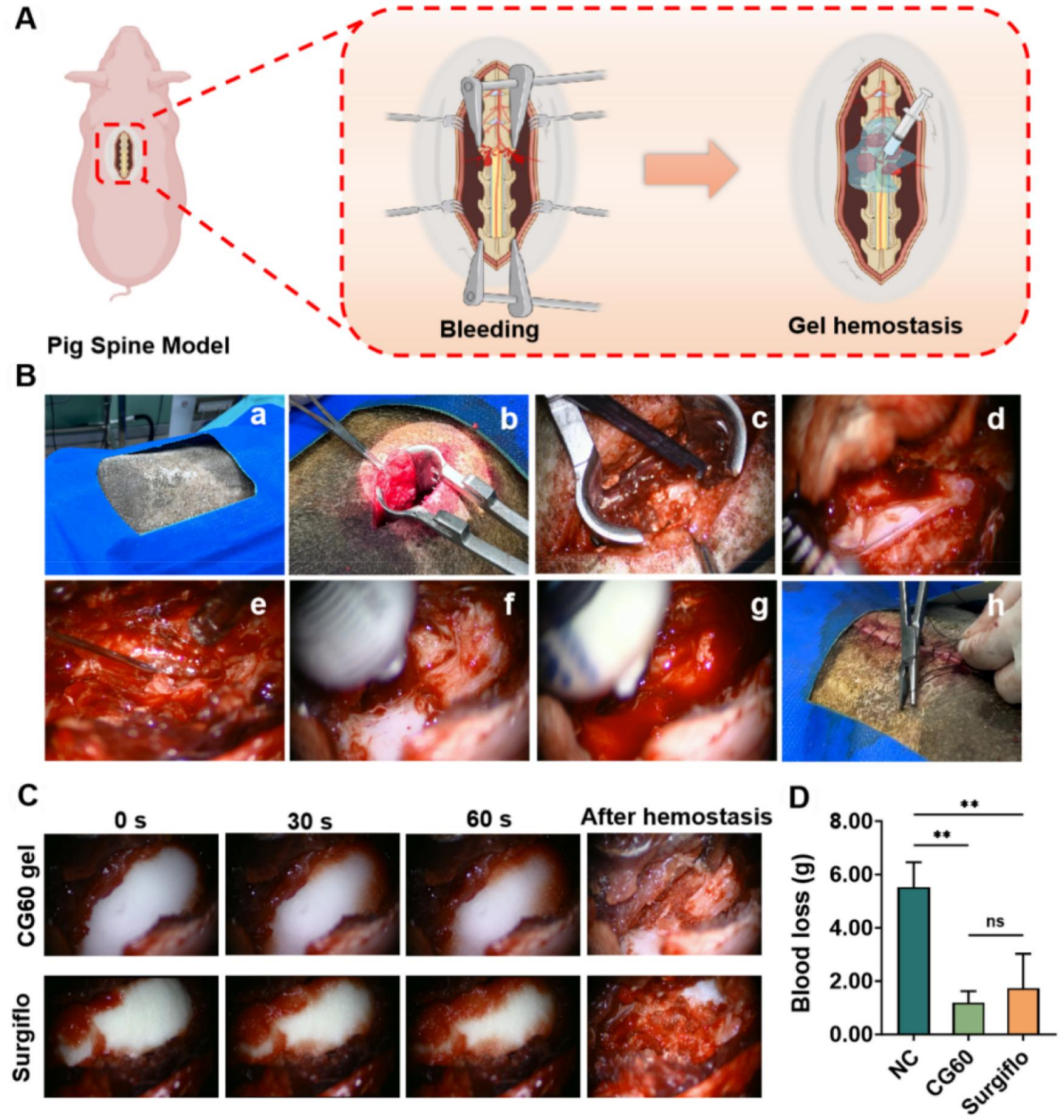
Hemostatic effect on non-compressible hemorrhage in pig spinal surgery. (**A**) Schematic diagram of the non-compressible hemorrhage model and the hemostat application in pig spinal surgery. (**B**) Photos of the surgery process: (**a**) conventional surgery area disinfection, draping sterile surgical towels; (**b, c**) cut open the skin, subcutaneous tissue, and lumbar fascia, and clean the soft tissue to expose the vertebral plate; (**d**) use bone-biting forceps to remove the vertebral plate and expose the spinal canal; (**e**) insert a syringe needle into the spinal canal, approach and cut the inner wall to prepare a bleeding model; (**f, g**) use CG60 gel and Surgiflo®, respectively, to stop bleeding; (**h**) disinfect and suture. (**C**) Photograph of pig spinal hemorrhage model with CG60 gel and Surgiflo®. (**D**) Blood loss with no treatment, CG60 gel, and Surgiflo®. Data are presented as mean ± SD. **P < 0.01, ns: not significant.

To assess the clinical translational potential of the CG, a paraspinal microvenous hemorrhage model was utilized to evaluate its hemostatic performance (Figure 5A and B). This model was designed to mimic clinically relevant moderate- to low-pressure, non-compressible bleeding scenarios encountered during spinal surgery, particularly venous plexus bleeding or cancellous bone surface oozing in deep, narrow operative fields. Such situations are challenging because direct compression is difficult and the bleeding site is often close to critical neural roots. Therefore, this model is particularly suitable for evaluating the practical value of injectable hemostatic materials in spinal surgery. Surgiflo®, a gelatin-based commercial hemostat widely employed in clinical practice [43], served as the control. Upon application to the bleeding site, the CG60 gel rapidly adhered to the wound surface via compressive forces, achieving hemostasis in 49 ± 3.61 s (Figure 5C and Supplementary Video S1). In contrast, untreated wounds exhibited persistent bleeding (>210 s; Supplementary Video S2), while Surgiflo®-treated wounds required 52 ± 9.17 s to arrest bleeding (Supplementary Video S3). Post-hemostasis, the CG60 gel demonstrated facile removal under saline irrigation, whereas Surgiflo® adhered rigidly to the wound cavity, posing risks of nerve compression due to persistent mechanical fixation. Total blood loss, quantified as the combined mass of blood absorbed by filter paper and retained within the hemostat, was significantly reduced in the CG60 gel group (1.20 ± 0.42 g) compared to untreated (5.52 ± 0.94 g) and Surgiflo®-treated (1.73 ± 1.29 g) groups (Figure 5D). The CG60 gel demonstrated superior efficacy in managing non-compressible spinal hemorrhage compared with both Surgiflo® and clinical gauze controls. This enhancement may be associated with synergistic electrostatic interactions between CMC and gelatin, a microporous architecture that facilitates platelet aggregation, and hydrophobic alkyl chains that enhance tissue adhesion [44]. In addition, to further explore the applicability boundary of the dual-form CG system under high-pressure arterial bleeding conditions, we supplemented the study with a rabbit carotid artery hemorrhage experiment (Supplementary Figure S3). In this high-flow arterial bleeding condition, CG60 powder provided more favorable bleeding control than gauze and Surgicel®, whereas the gel form was less suitable for this scenario, likely because rapid arterial flow may compromise local retention before stable barrier formation. These findings suggest that the two forms of CG60 have complementary hemostatic applicability. The gel form is more suitable for deep, narrow, and irregular spinal bleeding sites requiring conformal filling and local retention, whereas the powder form may be more advantageous for exposed high-flow arterial bleeding that requires rapid blood absorption and local clot formation.

### Biocompatibility and biodegradation of CG gel *in vivo*

**Figure 6:**
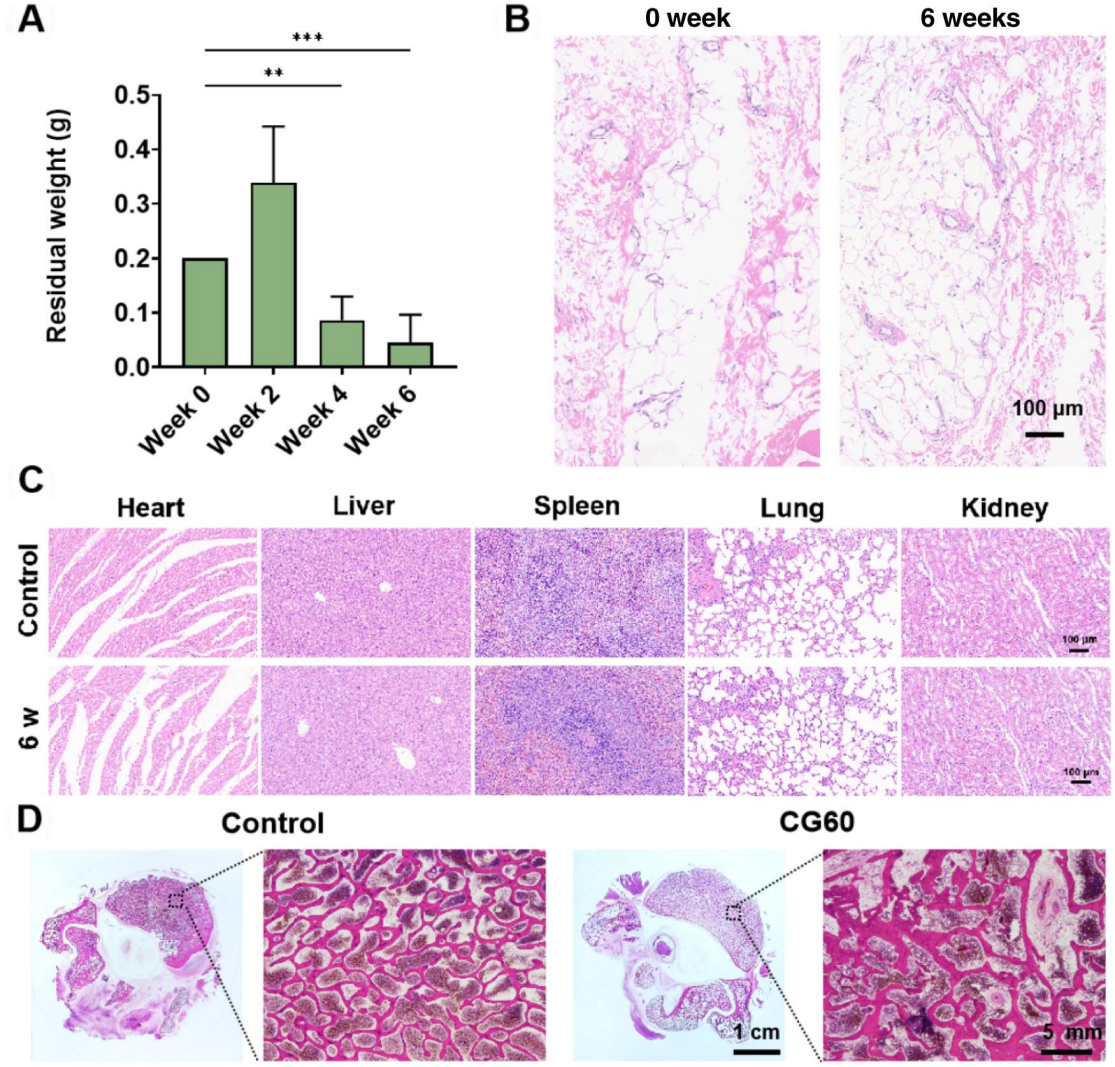
Biocompatibility and biodegradation of CG gel *in vivo*. (**A**) Weight of the CG60 gel implants after subcutaneous implantation for 2, 4 and 6 weeks (*n* = 3). (**B**) H&E staining of the implantation sites after implantation with the CG60 gel tokens for 6 weeks. (**C**) H&E staining of major organs (including heart, liver, spleen, lung and kidney) after treatment as indicated. Scale bar, 100 μm. (**D**) H&E staining of pig spine after hemostasis of CG60 gel at postoperative day 48. Data are presented as mean ± SD. **P < 0.01, ***P < 0.001, ns: not significant.

To evaluate the *in vivo* biodegradation of the CG60 gel, a subcutaneous implantation model was established in male SD rats. Specifically, 0.2 g of CG60 gel was injected subcutaneously into the back of rats. At 2, 4 and 6 weeks, the implantation sites were reopened to quantify the residual gel mass. As shown in Figure 6A, the mass of the implanted CG60 gel displayed a significant increase in the second week (0.39 ± 0.11 g), likely due to the absorption of surrounding tissue fluid and mild inflammatory exudation. Subsequently, a significant decrease was observed in the fourth week (0.09 ± 0.04 g). By the sixth week, the gel was nearly completely degraded (0.05 ± 0.05 g). These results confirm the CG60 gel’s capacity to maintain structural integrity within physiological environments while undergoing controlled degradation during hemostatic processes. It should be noted that the transient increase in CG60 gel mass in the subcutaneous implantation model suggests that the material can absorb surrounding tissue fluid during the early implantation stage. In the context of spinal surgery, such volume expansion deserves careful consideration because excessive postoperative swelling of a retained hemostatic material within the confined spinal canal could theoretically increase the risk of neural compression. However, the current subcutaneous model was designed to assess *in vivo* biodegradation rather than to simulate long-term retention in the spinal canal. In the intended surgical setting, CG60 gel is used as a topical hemostatic agent to rapidly control bleeding and can be removed by saline irrigation after hemostasis, thereby reducing the likelihood of persistent mass effect.

To evaluate *in vivo* biocompatibility, histopathological analysis was performed on tissues adjacent to implantation sites. H&E staining revealed no inflammatory cell infiltration in any experimental group (Figure 6B). Systemic biocompatibility was further assessed through H&E evaluation of major organs (heart, liver, spleen, lung, kidney), which showed comparable histoarchitecture pre- and post-implantation, with only minor localized macrophage/lymphocyte infiltration indicative of mild acute-phase responses (Figure 6C). As a novel spinal hemostatic material, the CG60 gel demonstrated superior biocompatibility in porcine model, as evidenced by comprehensive histopathological evaluation. Postoperative analysis through H&E staining revealed no discernible histopathological disparities between the CG60-treated group and control spinal tissue (Figure 6D). In contrast to the typical pathological features, no tissue vacuolization, structural disruption, or inflammatory responses were detected at the lumbar epicenter [45]. The bone tissue in both groups remained intact, with regular arrangement and normal staining of bone cells, and there were no signs of bone hyperplasia, destruction, or abnormal calcification. Additionally, no significant aggregation of inflammatory cells, such as neutrophils or lymphocytes, was observed in either group. This indicates that both the CG60 gel and the control groups were highly consistent across key indicators, such as bone structure and inflammatory response, suggesting that CG did not induce spinal degeneration, inflammation, or structural damage and thus exhibited excellent biocompatibility. The *in vivo* safety of CG60 was supported by both the subcutaneous implantation model and the porcine spinal histological evaluation. The former provided preliminary evidence of controlled biodegradation and acceptable local/systemic biocompatibility, while the latter directly suggested that topical application of CG60 in the spinal surgical field did not induce obvious structural damage or inflammatory abnormalities in adjacent tissues at the examined postoperative time point. Collectively, these findings demonstrate the excellent *in vivo* biocompatibility of the CG60 gel and its suitability for use in clinical applications.

Although the present results support preliminary *in vivo* biocompatibility, the current study did not include long-term implantation or spinal functional assessments. More comprehensive long-term safety evaluation, including neurological and functional outcomes, will be necessary in future studies to further support clinical translation.

## Conclusion

In summary, a novel dual-form (powder/injectable gel) hemostatic material was successfully developed by amidating gelatin and CMC to address uncontrollable bleeding in deep, narrow areas during spinal surgeries. The hemostatic performance was demonstrated *in vitro* and *in vivo*, with rapid blood coagulation and reduced blood loss. The gel form (CG60) performed particularly well in a pig spinal bleeding model, achieving rapid hemostasis and lower blood loss than in untreated groups. Biocompatibility experiments confirmed its bio-safety *in vivo*. Overall, the present study demonstrates promising preclinical potential of the dual-form CG system as a hemostatic material for managing non-compressible bleeding in spinal surgery.

## Supplementary Material

rbag135_Supplementary_Data
