# Supplementary material for: Dual-mode rapid hemostatic materials composed of gelatin and carboxymethyl cellulose for non-compressible bleeding in spinal surgery
Source: Regen Biomater. 2026 Jun 16;13:rbag135. doi: 10.1093/rb/rbag135 (PMC13354601; doi:10.1093/rb/rbag135)
Supplement: rbag135_Supplementary_Data [file rbag135_supplementary_data.zip › Supplementary Material.docx]

**Dual-mode rapid hemostatic materials composed of gelatin and carboxymethyl cellulose for non-compressible bleeding in spinal surgery**

Xiaoting Peng ^#a^, Mingze Ji ^#b^, Bingcheng Fan ^#b^, Chuqiang Yin ^c^, Ting Wang *^c^, Jianyong Du *^a^, Guotai Li *^a^, Qihui Zhou *^a^

Author Affiliations:

^a^ Qingdao Municipal Hospital, Shandong Key Laboratory of Neurorehabilitation, Shandong Engineering Research Center for Tissue Rehabilitation Materials and Devices, Qingdao Key Laboratory of Smart Rehabilitation Material, School of Rehabilitation Sciences and Engineering, University of Health and Rehabilitation Sciences, Qingdao 266113, China.

^b^ College of Medicine, Qingdao University, Qingdao 266071, China.

^c^ Department of Orthopaedic Surgery, The Affiliated Hospital of Qingdao University, Qingdao 266003, China.

# These authors contributed equally to this work.

*Corresponding author:

Ting Wang, M.D., Professor, Email: tingwang@qdu.edu.cn.

Jianyong Du, Ph.D., Associate Professor, Email: dujianyong66@uhrs.edu.cn.

Guotai Li, Ph.D., Associate Professor, Email: liguotai@uhrs.edu.cn.

Qihui Zhou (周祺惠), Ph.D., Professor, Phone: +86-17660670299, Email: qihuizhou@uhrs.edu.cn.


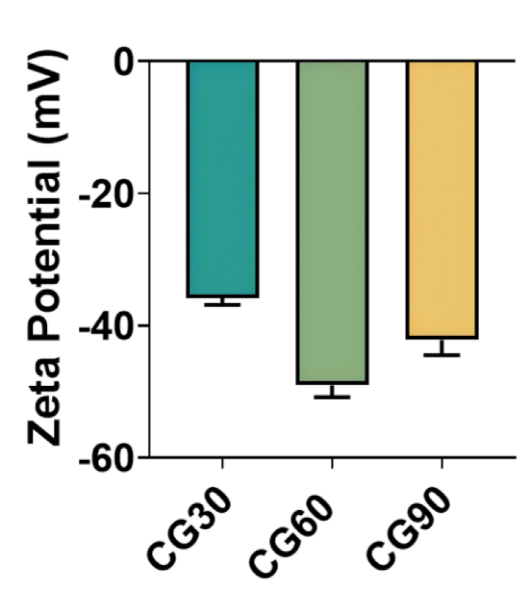


**Fig. S1.** The zeta potential measurements for CG30, CG60, and CG90.

**Fig. S2.** Compressive stress-strain curves for CG30, CG60, CG90, and Surgiflo^®^.

**
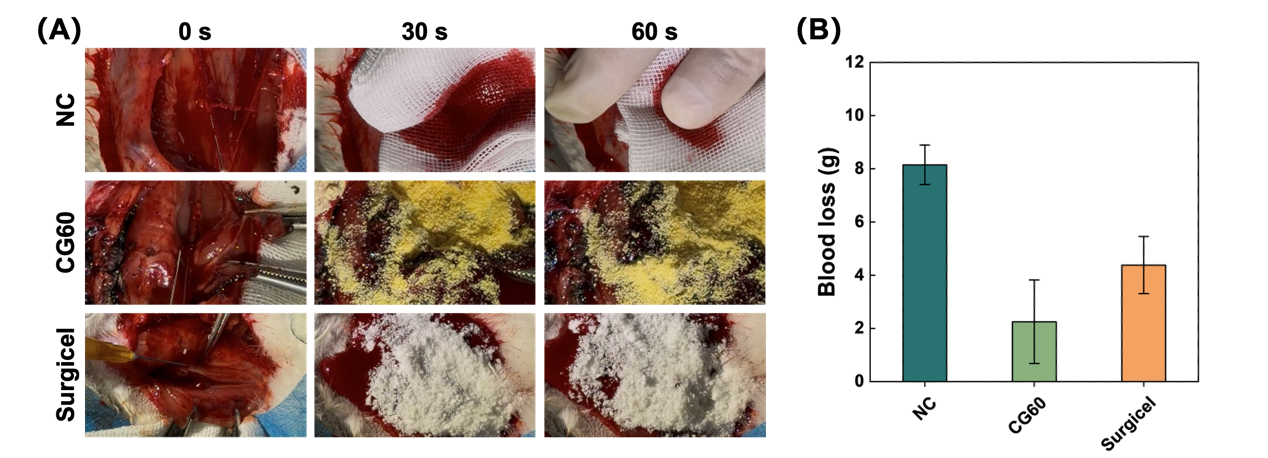
**

**Fig. S3.** Hemostatic evaluation of the dual-form CG system in a rabbit carotid artery hemorrhage model. (A) Representative photographs of bleeding control in the gauze, Surgicel®, and CG60 powder groups. (B) Quantitative analysis of blood loss in different treatment groups.


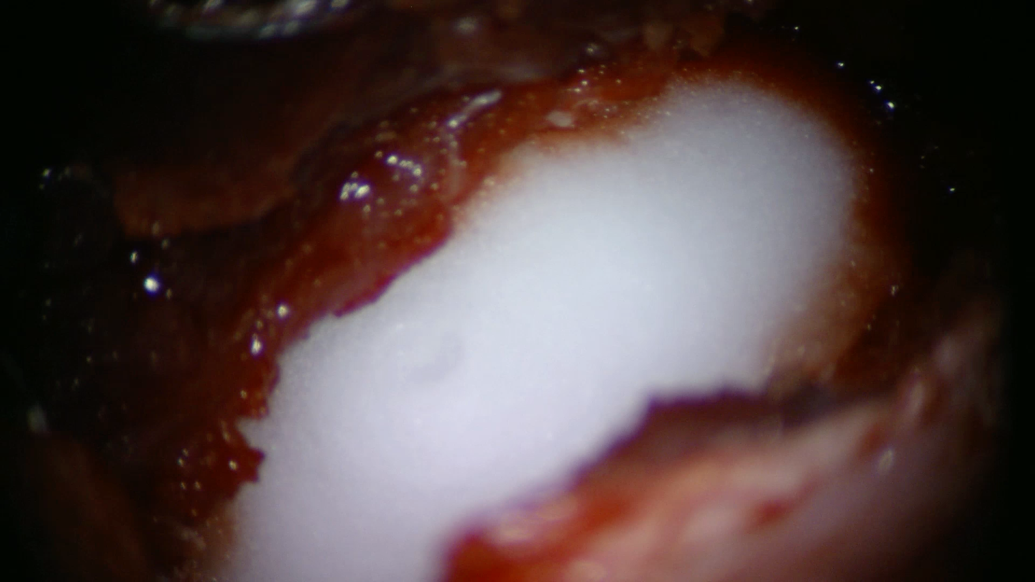


**Supporting Video 1.** Hemostatic process of CG60 gel in the porcine paraspinal microvenous hemorrhage model, showing rapid local adhesion and bleeding control; Following incision of the spinal canal wall with a syringe needle, 300 μL of CG60 gel was immediately injected. The video shows rapid blood absorption, and the hemostasis time was determined by the rapid stabilization of the bleeding area.


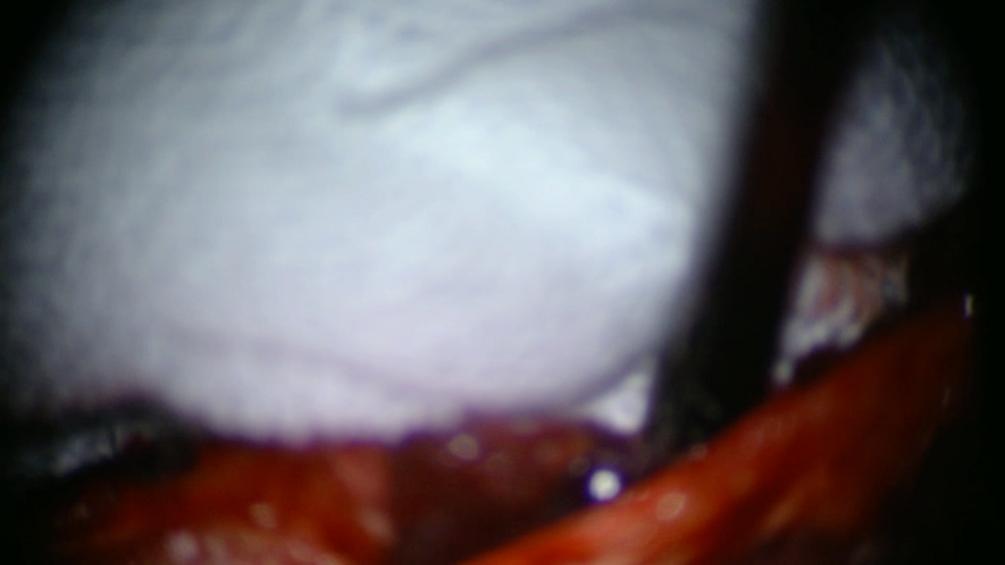


**Supporting Video 2.** Untreated bleeding in the porcine model, showing persistent hemorrhage without effective hemostasis. Following incision of the spinal canal wall with a syringe needle, gauze was immediately applied to the bleeding site. Compared with the CG60 gel, the gauze failed to adequately fill the narrow and irregular cavity to achieve sufficient compression or absorb the exudate, and hemostasis was not achieved even after a prolonged period.


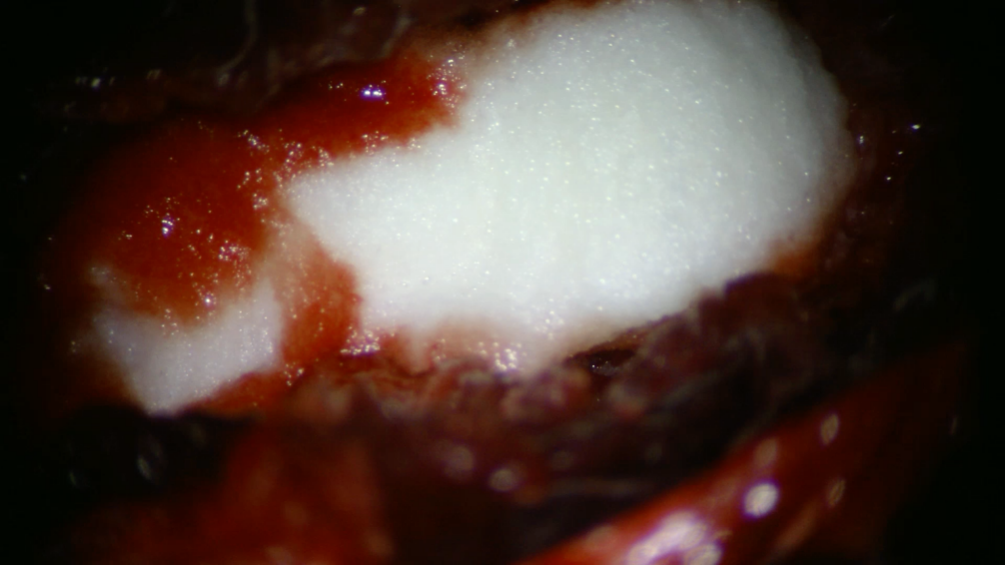


**Supporting Video 3.** Hemostatic process of Surgiflo^®^ in the porcine model, showing bleeding control under the same surgical setting. After injection of the same volume (300 μL) of Surgiflo^®^, it also exhibits rapid blood absorption and cavity filling, but the stabilization time is slightly longer than that of CG60 gel.
